# Financial incentive strategies for maintenance of weight loss: results from an internet-based randomized controlled trial

**DOI:** 10.1038/s41387-018-0036-y

**Published:** 2018-05-25

**Authors:** William S. Yancy Jr., Pamela A. Shaw, Lisa Wesby, Victoria Hilbert, Lin Yang, Jingsan Zhu, Andrea Troxel, David Huffman, Gary D. Foster, Alexis C. Wojtanowski, Kevin G. Volpp

**Affiliations:** 1 Duke University Diet and Fitness Center, Durham, NC USA; 2 Department of Medicine, Duke University School of Medicine, Durham, NC USA; 3 Department of Veterans Affairs, Center for Health Services Research in Primary Care, Durham, NC USA; 4 Department of Biostatistics, Epidemiology and Informatics, Perelman School of Medicine, University of Pennsylvania, Philadelphia, PA USA; 5 Leonard Davis Institute Center for Health Incentives and Behavioral Economics, Perelman School of Medicine, University of Pennsylvania, Philadelphia, PA USA; 6 Department of Health Care Management, The Wharton School, University of Pennsylvania, Philadelphia, PA USA; 7 Center for Clinical Epidemiology and Biostatistics, Perelman School of Medicine, University of Pennsylvania, Philadelphia, PA USA; 8 Department of Medical Ethics and Health Policy, Perelman School of Medicine, University of Pennsylvania, Philadelphia, PA USA; 9 Department of Medicine, Perelman School of Medicine, University of Pennsylvania, Philadelphia, PA USA; 10 Department of Population Health, New York University School of Medicine, New York, NY USA; 11 Department of Economics, University of Pittsburgh, Pittsburgh, PA USA; 12 Department of Science and Innovation, Weight Watchers International, New York, NY USA; 13 Center for Obesity Research and Education, Temple University, Philadelphia, PA USA; 14 Center for Weight and Eating Disorders, University of Pennsylvania, Philadelphia, PA USA; 15 Center for Health Equity Research and Promotion, Philadelphia Veterans Affairs Medical Center, Philadelphia, PA USA

## Abstract

**Background/objective:**

Financial incentives can improve initial weight loss; we examined whether financial incentives can improve weight loss maintenance.

**Subjects/methods:**

Participants aged 30–80 years who lost at least 5 kg during the first 4–6 months in a nationally available commercial weight loss program were recruited via the internet into a three-arm randomized trial of two types of financial incentives versus active control during months 1–6 (Phase I) followed by passive monitoring during months 7–12 (Phase II). Interventions were daily self-weighing and text messaging feedback alone (control) or combined with a lottery-based incentive or a direct incentive. The primary outcome was weight change 6 months after initial weight loss. Secondary outcomes included weight change 12 months after initial weight loss (6 months after cessation of maintenance intervention), and self-reported physical activity and eating behaviors.

**Results:**

Of 191 participants randomized, the mean age was 49.0 (SD = 10.5) years and weight loss prior to randomization was 11.4 (4.7) kg; 92% were women and 89% were White. Mean weight changes during the next 6 months (Phase I) were: lottery −3.0 (5.8) kg; direct −2.8 (5.8) kg; and control −1.4 (5.8) kg (all pairwise comparisons *p* > 0.1). Weight changes through the end of 12 months post-weight loss (Phase II) were: lottery −1.8 (10.5) kg; direct −0.7 (10.7) kg; and control −0.3 (9.4) kg (all pairwise comparisons *p* > 0.1). The percentages of participants who maintained their weight loss (defined as gaining ≤1.36 kg) were: lottery 79%, direct 76%, and control 67% at 6 months and lottery 66%, direct 62%, and control 59% at 12 months (all pairwise comparisons *p* > 0.1). At 6 and 12 months after initial weight loss, changes in self-reported physical activity or eating behaviors did not differ across arms.

**Conclusions:**

Compared with the active control of daily texting based on daily home weighing, lottery-based and direct monetary incentives provided no additional benefit for weight loss maintenance.

## Introduction

A number of strategies have been successful in achieving initial weight loss, but maintenance of weight loss has consistently been more challenging^1–3^. A wide range of factors may contribute to this challenge, including changes in resting metabolic rate and fundamentally different behavioral processes that are active in weight loss maintenance versus weight loss such as goal, duration, motivation, adherence, cost versus benefit, role of activity, and reinforcement^4–7^.

An external motivational source such as monetary incentives can be effective in inducing initial weight loss^8^. Research has shown that variable reinforcement using lottery payments may be more effective over time than a fixed incentive, and frequent incentives are more effective than infrequent incentives for behavior change^9^. Given that weight loss maintenance is arguably considerably more challenging, however, it is uncertain whether financial incentives will be effective in this context. This study compared the efficacy of two daily monetary incentive strategies (lottery-based or a direct incentive) versus an active control condition (daily self-weighing with text message feedback) for weight loss maintenance over 12 months after initial weight loss.

## METHODS

### Overview of study design

This three-arm randomized controlled trial had two phases after initial weight loss^10^. The study protocol is available in Supplement 1. In Phase I, participants received one of three interventions for 6 months: (1) daily self-weighing and text messaging feedback (control), (2) daily self-weighing and text messaging feedback combined with direct monetary incentive (direct payment), or (3) daily self-weighing and text messaging feedback combined with a lottery-based monetary incentive (lottery). In Phase II (months 7–12 after initial weight loss), all participants were observed without intervention for 6 additional months. At the end of Phase I, participants were encouraged to continue weighing themselves daily (during Phase II), but text messaging feedback and incentives ceased.

### Participants and setting

Participants were recruited from September 2013 to June 2014 using a nationally available weight loss program, Weight Watchers (WW). WW members who had opted to receive email communication from Weight Watchers International, Inc. and met the eligibility criteria were sent an email inviting participation in the study. In the email, a link transferred potential participants to the Way to Health portal, a web-based platform based at the University of Pennsylvania that integrates clinical trial enrollment and randomization processes, wireless devices (such as scales), messaging (text, email, or voice), self-administered surveys, and distribution of financial incentives^11^. We recruited from WW centers (*n* = 505) across 41 states that were able to electronically transmit in-person weight measurements to a WW coordinating site, allowing verification of self-reported weights. Eligibility criteria were ages 30–80 years, body mass index (BMI) 30– 45 kg/m^2^ prior to starting WW, documented weight loss of at least 5 kg in the first 4–6 months on the WW meetings plus digital tools (Monthly Pass) program, active WW meetings membership, reliable access to the internet, and a smartphone that could be paired with a wireless scale. Exclusion criteria were substance abuse; bulimia nervosa or related behaviors; pregnancy or breast feeding; medical contraindications to counseling about diet, physical activity, or weight reduction; unstable mental illness; and positive screen for pathologic gambling.

Informed consent was obtained from all participants via the Way to Health portal (https://www.waytohealth.org/). Once baseline weight was verified by WW staff, participants completed a survey online, received a wireless scale (Withings Corp., Issy-les-Moulineaux, France), and, after their first weight transmission, were randomized and notified of their arm assignment via the portal with allocation concealed from study staff until this point. Computer-generated randomization occurred in a 2:2:1 ratio for the interventions versus control during rolling enrollment using variable block sizes of 5 and 10 and stratification by sex and baseline BMI (BMI 30–37.9 and 38–45 kg/m^2^). The 2:2:1 ratio allowed for adequate power to detect a difference between the two financial incentive intervention arms, which was pre-hypothesized to be smaller than the difference between each financial incentive and control.

### Interventions

Each participant selected a personal weekly weight goal of 0, −0.5, or −1 lb, which could be reset by the participant monthly if desired to allow personalization of goals. We chose this design because we expected some participants would reach their goal weight during the study, whereas other participants would desire further weight loss. If at any time weight increased such that the participant was above the initial weight at the start of the study, then maintaining weight (goal of 0 lb) was not an allowable option. Participants were asked to weigh themselves every morning in minimal clothing before eating or drinking and after urinating. The weight result was transmitted wirelessly to the study database triggering a daily text message on progress relative to the chosen weight goal, with additional messages about monetary winnings for those participants. Daily messaging was chosen as the control condition to standardize the type and frequency of participant feedback in order to examine the incremental impact of the incentives because the daily incentives required messaging on a daily basis. Weight measurements were verified in-person at a WW location at months 3, 6, 9, and 12.

Participants were considered at goal each day their transmitted weight was equal to or less than their goal weight for that week. Participants in the direct payment condition were eligible to receive US$2.80 each day their transmitted weight was at goal. For example, a participant who chose a weekly goal of −0.5 lb a week and succeeded in achieving their goal weight each day for 12 weeks would earn US$2.80 for 7 days a week for 12 weeks for a total of US$235. Participants in the lottery condition were eligible for a chance to win a daily prize each day their transmitted weight was at goal. Eligible lottery participants had an 18 in 100 chance of winning US$10 and 1 in 100 chance of winning US$100 for an expected value of US$2.80 per day. For example, a participant who chose a weekly goal of −0.5 lb a week and remained at goal weight each day for 12 weeks might win the small lottery 14 times and the large lottery once for a total of US$240. If a participant’s lottery number was chosen but the participant did not transmit a weight or weight was above goal that day, a text message was sent indicating that the participant would have won the lottery if requirements had been met, an approach designed to induce anticipated regret and increase motivation^8^. The monetary amount of US$2.80 per day was based on prior research demonstrating efficacy for weight loss^8^.

In both incentive conditions, participants received their winnings via check every 3 months. The amount participants actually received depended on their in-person weight measurement at 3 and 6 months, respectively, relative to their goals. In other words, a participant 100% of the way toward the goal received the full winnings, but a participant 50% toward the goal received 50% of the winnings. For the direct arm example participant above who chose the −0.5 lb goal for 12 weeks, the expected weight loss would be 6 lbs; if the participant’s in-person weight at the 3-month time point was only down 4 lbs, the participant would receive 4/6ths or 75% x US$135 = US$101.25. All participants were allowed to keep the scales and were compensated up to US$160 for participation in the study (US$30 for the visit at 3 and 9 months; US$50 for the visit at 6 and 12 months).

### Measurements

The primary outcome was change in weight from study enrollment (which was after initial weight loss in WW) to 6 months using the in-person weight (shoes and heavy items removed) from WW locations. WW staff members at these locations were not part of the study staff nor made aware of participant treatment assignment. Study staff members were not involved in collection of these measurements. A key secondary outcome was change in weight 12 months after initial weight loss. Participants also completed a web-based questionnaire at these time points. Physical activity was measured using the International Physical Activity Questionnaire (IPAQ)-Long^12^. Eating habits were assessed using the Three-factor Eating Questionnaire-R18^13^.

### Safety monitoring

The Institutional Review Boards of the University of Pennsylvania and Duke University approved the study. The study was also monitored by an independent Data Safety Monitoring Board^10^. Daily weight data were used to screen for excessive weight loss. Participants were contacted if they lost >7 lbs in 1 week or >12 lbs in 1 month and asked about potential unsafe efforts to lose weight.

### Statistical methods

The primary objective was to examine weight changes 6 months after initial weight loss (Phase I) in the following pairwise comparisons: (1) the daily lottery-based financial incentive versus control, (2) the direct payment incentive versus control, and (3) the lottery financial incentive versus direct payment financial incentive. A key secondary objective was to compare weight changes 12 months after initial weight loss or 6 months following the cessation of the interventions (Phase II).

**Fig. 1:**
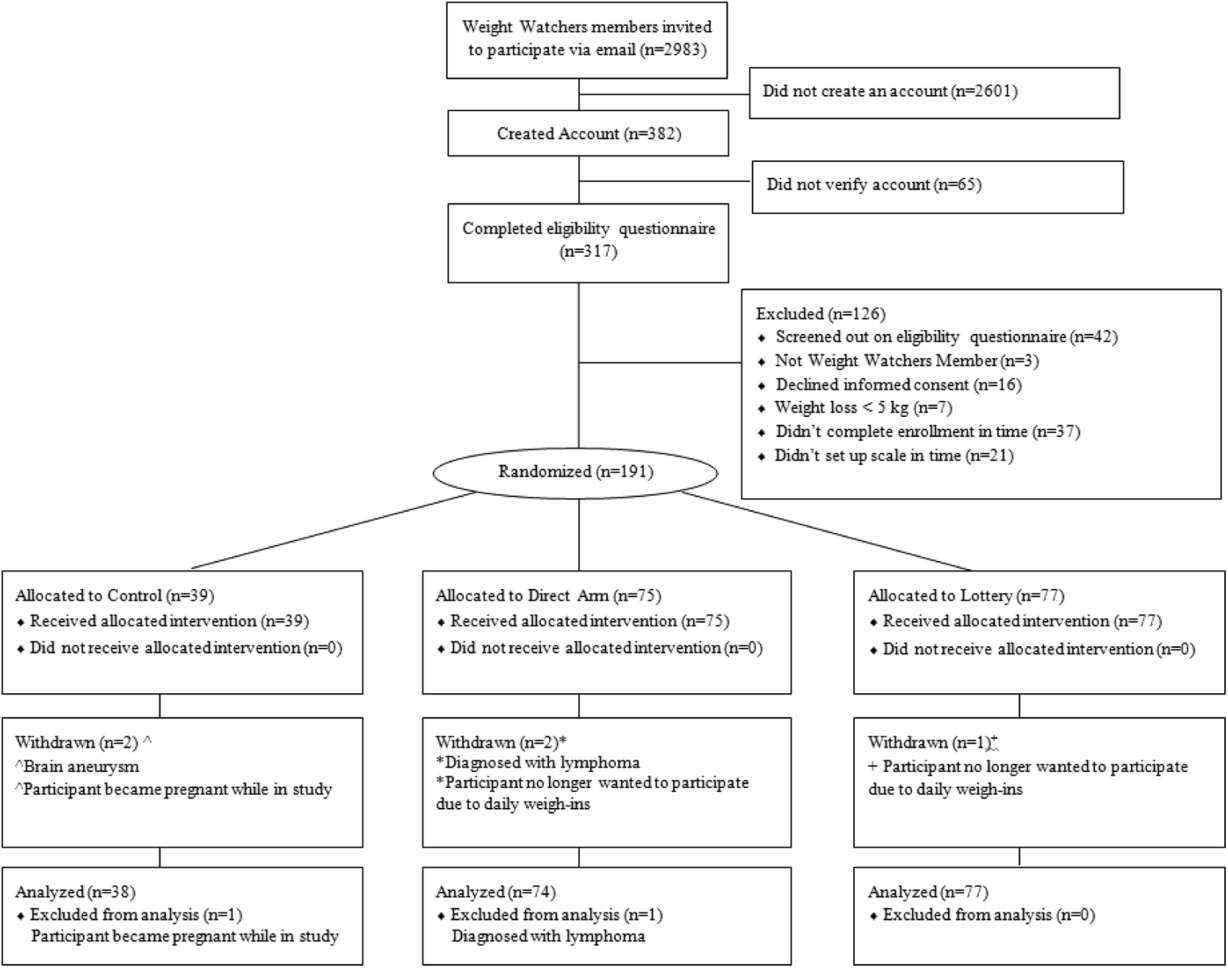
**Flow of study participants**

All primary and secondary analyses used a modified intent-to-treat strategy, excluding two participants found to have exclusion criteria after randomization (Fig. 1). Missing in-person weight data were multiply imputed using linear regression adjusted for baseline BMI, baseline weight, weight loss amount in WW prior to randomization, weight loss goal chosen the first week of the study, study arm, and participant demographics^14^. Sensitivity analyses were performed using a multiple imputation strategy that additionally used post baseline information on weight, subsequent weight loss goals, and WW continued membership, and a single imputation strategy that assumed that participants with missing weight outcome returned to their baseline weight^15^. Complete case analyses were also performed, using no adjustment for missing data, as a per-protocol approach. A multivariable regression analysis of the primary outcome was performed to obtain an arm comparison adjusted for the baseline factors age, sex, race, education, income, weight, WW program starting BMI, and qualifying weight loss. We calculated the percentage of participants in each group who maintained their weight loss and defined maintenance as gaining <1.36 kg (3.0 lbs), which corresponds to <2% body fluid volume change in a wide range of body weights^16^. A post hoc analysis of the frequency of at-home weight measurements compared arms using a generalized estimating equation with an autoregressive-1 working correlation model; mean number of days out of 7 was compared among study arms adjusting for study week and a week-by-time interaction. Separate models were fit for Phases 1 and 2. A linear mixed-effects model with random intercept and slope for week was also considered. Significance tests were two-sided and unless otherwise stated done at the 0.05 level. Analyses were performed in SAS (version 9.4; SAS Institute) and R software (version 3.4.0; R Development Core Team, Vienna, Austria).

Sample size was estimated allowing for the Holm–Bonferroni method to sequentially test the three primary comparisons maintaining an *α* of 0.05 and a power of 90%^17^. Based on data from our prior study, a sample of *N* = 150 was needed to detect a difference in weight change during Phase I of 5 kg between each incentive group and the control group and 3 kg between incentive groups, assuming a standard deviation in weight change of 5 kg^8^. We estimated loss to follow-up of 20%, resulting in a final target sample size of *N* = 188 participants.

## RESULTS

### Recruitment and enrollment

**Fig. 2:**
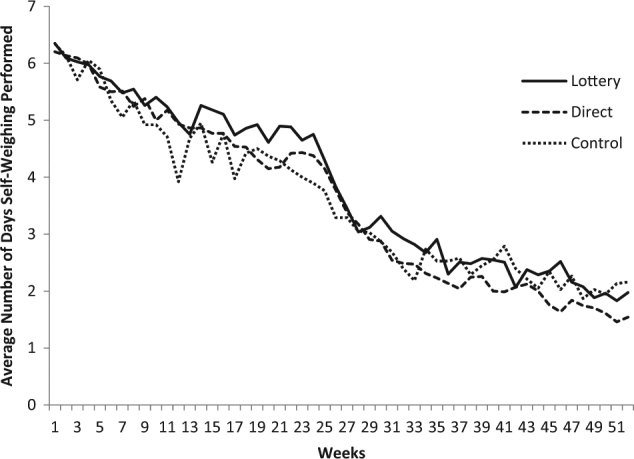
**Average number of days per week self-weighing was performed by arm over time (randomization is time 0)**

A total of 2983 WW members received an invitation email and 191 participants were enrolled (Fig. 1). The mean (SD) age of the participants was 49.0 (10.5) years; 92% were women, 89% were White, and 62% had at least a college degree (Table 1). The mean measured weight upon starting WW was 101.6 (15.7) kg and at study enrollment was 90.2 (14.9) kg for a mean weight loss prior to study enrollment of 11.4 (4.7) kg or 11.2% of original body weight. Mean BMI at randomization was 32.5 (4.1) kg/m^2^. In-person weight measurements were available at 6 months after initial weight loss for 90.9% of lottery, 89.2% of direct, and 86.8% of control participants, and at 12 months for 81.8% (lottery), 75.7% (direct), and 81.6% (control). Participants weighed themselves at home approximately 90% of days in the first week, 65% of days in week 26, and 30% of days during the last week of follow-up, with no statistically significant differences in these patterns over time across arms (*p* value for interaction between arm and study week: Phase 1: *p* = 0.508; Phase 2: *p* = 0.310; Fig. 2).

**Table 1:** Baseline characteristics of study participants overall and by arm

Characteristic	Total (*n* = 191)	Lottery (*n* = 77)	Direct payment (*n* = 75)	Control (*n* = 39)
Age, mean (SD)	49.0 (10.5)	48.8 (10.3)	48.6 (11.0)	50.1 (10.0)
Female gender, *n* (%)	175 (91.6)	70 (90.9)	69 (92.0)	36 (92.3)
Race, *n* (%)				
White	170 (89.0)	68 (88.3)	69 (92.0)	33 (84.6)
Black	8 (4.2)	3 (3.9)	4 (5.3)	1 (2.6)
Other	6 (3.1)	3 (3.9)	1 (1.3)	2 (5.1)
Two or more races	7 (3.7)	3 (3.9)	1 (1.3)	3 (7.7)
Hispanic, *n* (%)	8 (4.2)	2 (2.6)	4 (5.3)	2 (5.1)
Education, *n* (%)				
Less than college	72 (37.7)	26 (33.8)	35 (46.7)	11 (28.2)
College graduate	55 (28.8)	28 (36.4)	11 (14.7)	16 (41.0)
Post-college degree	64 (33.5)	23 (29.9)	29 (38.7)	12 (30.8)
Household income, *n* (%)				
<US$50,000	23 (12.0)	12 (15.6)	5 (6.7)	6 (15.4)
US$50,000 to <US$100,000	96 (50.3)	36 (46.8)	40 (53.3)	20 (51.3)
≥US$100,000	72 (37.7)	29 (37.7)	30 (40.0)	13 (33.3)
People per household, mean (SD)	3.1 (1.4)	3.2 (1.5)	3.0 (1.4)	3.0 (1.2)
Weight measures, mean (SD)				
Weight at the start of Weight Watchers	101.6 (15.7)	100.8 (15.7)	101.7 (16.0)	102.9 (15.6)
Weight at randomization	90.2 (14.9)	89.2 (14.3)	90.7 (15.7)	91.2 (15.0)
Weight loss prior to randomization	11.4 (4.7)	11.6 (4.4)	10.9 (5.1)	11.7 (4.3)
BMI measures				
BMI at start of Weight Watchers, mean (SD)	36.7 (4.3)	36.5 (4.4)	36.8 (4.1)	36.9 (4.5)
BMI at randomization, mean (SD)	32.5 (4.1)	32.3 (4.1)	32.8 (4.3)	32.6 (4.1)
BMI ≥35 kg/m^2^ at randomization, *n* (%)	69 (36.1)	26 (33.8)	29 (38.7)	14 (35.9)
Eating behaviors by TFEQ, mean (SD)^a^				
Cognitive restraint scale	62.3 (14.4)	62.0 (15.0)	62.6 (14.6)	62.4 (12.8)
Uncontrolled eating scale	45.1 (16.0)	44.4 (15.6)	47.6 (16.2)	41.8 (16.1)
Emotional eating scale	59.9 (24.5)	58.7 (23.8)	63.3 (23.5)	55.8 (27.2)
Physical activity in min per week^b^, median (IQR)				
Moderate-vigorous activity min per week	333 (120, 700)	345 (180, 885)	240 (60, 585)	390 (180, 630)
Walking min per week	148 (45, 428)	180 (53, 537.5)	100 (20, 360)	150 (90, 325)
Total activity min per week	510 (238, 1148)	533 (283, 1295)	460 (150, 990)	600 (270, 925)

^a^ *TFEQ* Three-Factor Eating Questionnaire. The raw eating scale scores are transformed to a 0–100 scale [((raw score − lowest possible raw score)/possible raw score range) × 100] Higher scores in the respective scales are indicative of greater cognitive restraint, uncontrolled, or emotional eating

^b^ Measured using the International Physical Activity Questionnaire. Data were missing for three participants: Lottery (*n* = 1), Direct (*n* = 2)

### Weight outcome

**Fig. 3:**
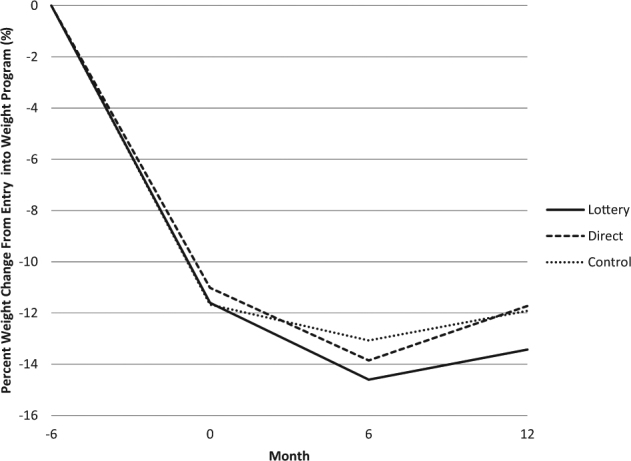
**Percent weight change by arm from entry into Weight Watchers program over time (randomization is time 0)**

For the primary outcome, maintenance of weight loss occurred across all arms (Fig. 3). Mean (SD) weight changes at 6 months after initial weight loss were: lottery −3.0 (5.8) kg; direct −2.8 (5.8) kg; and control −1.4 (5.8) kg (all pairwise comparisons *p* > 0.1) (Table 2 and Fig. 3). Mean weight changes at 12 months after initial weight loss were: lottery −1.8 (10.5) kg; direct −0.7 (10.7) kg; and control −0.2 (9.4) kg (all pairwise comparisons *p* > 0.1). Within arms, weight change (additional weight loss) was statistically significant from baseline to 6 months in the lottery (*p* < 0.001) and direct (*p* < 0.001) incentive arms but not the control arm (*p* > 0.1). However, weight change was not statistically significant in any of the three arms at 12 months (*p* > 0.1). The percentages of participants who maintained their weight (defined as gaining no more than 1.36 kg or 3 lbs) at 6 months were lottery 79%, direct 76%, and control 67% (all pairwise comparisons *p* > 0.1) and at 12 months were lottery 66%, direct 62%, and control 59% (all pairwise comparisons *p* > 0.1). There was a trend across arms that weight loss was greater in those who weighed themselves more frequently (eFigure 4 in Supplement 2; *p* < 0.001 at 6 and 12 months). Adjusted models indicated that results were qualitatively similar after adjusting for factors that were measured at baseline. Sensitivity analyses for the primary outcome revealed very similar results across all imputation strategies.

**Table 2:** Imputed change in weight, physical activity, and eating behaviors at 6 and 12 months by arm

	Lottery (*n* = 77)	Direct payment (*n* = 74)	Control (*n* = 38)	Lottery versus control	Direct payment versus control	Lottery versus direct payment
Imputed weight change (kg)^a^						
6-month imputed weight change, mean (SD)	−3.0 (5.8)	−2.8 (5.8)	−1.4 (5.8)	−1.6	−1.4	−0.2
95% CI				−3.8, 0.6	−3.7, 0.8	−2.0, 1.7
* P* value				0.154	0.215	0.859
12-month imputed weight change, mean (SD)	−1.8 (10.5)	−0.7 (10.7)	−0.2 (9.4)	−1.6	−0.5	−1.1
95% CI				−5.3, 2.2	−4.3, 3.4	−4.3, 2.1
*P* value				0.408	0.813	0.494
Moderate-vigorous activity min per week						
6-month change, mean (SD)	50.5 (631.9)	−27.9 (529.3)	270.8 (610.6)	−220.4	−298.8	78.4
95% CI				−535.2, 94.5	−624.7, 27.2	−166.9, 323.7
*P* value				0.168	0.072	0.528
12-month change, mean (SD)	−15.2 (600.3)	89.6 (376.3)	101.3 (414.1)	−116.4	−11.7	−104.7
95% CI				−409.7, 176.9	−298.0, 274.6	−338.2, 128.7
*P* value				0.432	0.936	0.375
Walking min per week						
6-month change, mean (SD)	122.3 (492.2)	23.5 (445.5)	−18.7 (295.0)	141	42.2	98.7
95% CI				−96.6, 378.5	−204.3, 288.8	−86.0, 283.5
*P* value				0.242	0.735	0.292
12-month change, mean (SD)	−0.2 (540.2)	68.3 (236.0)	127.2 (459.2)	−127.3	−58.9	−68.5
95% CI				−382.4, 127.7	−307.8, 190.1	−271.5, 134.5
*P* value				0.323	0.639	0.504
Total activity min per week						
6-month change, mean (SD)	171.0 (908.2)	−4.4 (793.7)	252.2 (776.8)	−81.2	−256.5	175.4
95% CI				−530.1, 367.7	−722.5, 209.4	−173.8, 524.5
*P* value				0.721	0.278	0.322
12-month change, mean (SD)	−15.3 (932.0)	157.9 (435.1)	228.4 (670.2)	−243.8	−70.5	−173.2
95% CI				−673.8, 186.3	−490.3, 349.2	−515.5, 169.1
*P* value				0.263	0.739	0.317
Cognitive restraint scale, TFEQ						
6-month scale change, mean (SD)	1.5 (17.4)	3.6 (12.2)	3.2 (18.2)	−1.8	0.4	−2.2
95% CI				−10.1, 6.6	−8.2, 9.0	−8.6, 4.3
*P* value				0.679	0.927	0.508
12-month scale change, mean (SD)	−2.0 (17.9)	−2.2 (16.2)	2.8 (16.9)	−4.8	−4.9	0.1
95% CI				−15.1, 5.5	−15.1, 5.2	−8.0, 8.3
*P* value				0.358	0.337	0.973
Uncontrolled eating scale, TFEQ						
6-month scale change, mean (SD)	−3.5 (13.8)	0.3 (16.1)	−2.1 (15.7)	−1.4	2.4	−3.8
95% CI				−9.3, 6.6	−5.8, 10.6	−9.9, 2.4
*P* value				0.738	0.563	0.226
12-month scale change, mean (SD)	−3.7 (15.6)	0.5 (11.9)	−3.2 (14.9)	−0.5	3.8	−4.2
95% CI				−8.9, 8.0	−4.6, 12.1	−10.9, 2.5
*P* value				0.914	0.374	0.214
Emotional eating scale, TFEQ						
6-month scale change, mean (SD)	−6.7 (16.5)	−1.3 (18.2)	−4.7 (16.3)	−2.0	3.4	−5.4
95% CI				−11.1, 7.0	−6.0, 12.7	−12.4, 1.5
*P* value				0.658	0.474	0.126
12-month scale change, mean (SD)	−4.4 (19.4)	0.0 (14.8)	3.5 (15.0)	−7.9	−3.5	−4.4
95% CI				−18.0, 2.3	−13.5, 6.6	−12.4, 3.7
*P* value				0.129	0.493	0.283

*TFEQ* Three-Factor Eating Questionnaire. The raw eating scale scores are transformed to a 0–100 scale [((raw score − lowest possible raw score)/possible raw score range) × 100] Higher scores in the respective scales are indicative of greater cognitive restraint, uncontrolled, or emotional eating

^a^Weight change (primary outcome) models were adjusted for the baseline factors age, sex, race, education, income, weight, WW program starting BMI, and qualifying weight loss

### Secondary outcomes

At 6 and 12 months after initial weight loss, changes in self-reported physical activity or in the three domains of eating behaviors (cognitive restraint, uncontrolled eating, emotional eating) were not statistically significantly different across arms (Table 2).

Over the 6 months of the weight loss maintenance intervention, 98 incentive payments (total of US$10,056.00) were made to direct incentive participants (mean (SD) US$134.09 (US$125.26), maximum US$453.60, and minimum US$0.00) and 98 payments (total of US$11,901.00) were made to lottery incentive participants (mean US$154.56 (US$186.18), maximum US$590.00, and minimum US$0.00). The total potential winnings (i.e., had participants fully met their weight loss goals at 3 and 6 months by maintaining their transmitted at-home weight until the in-person verified weight) were US$13,090 for direct participants and US$15,500 for lottery participants. Lottery participants won US$10 a mean of 14.1 times and US$100 a mean of 0.8 times over the 180 days of the active phase of the intervention.

### Excess weight loss events and other adverse events

A total of 185 weight loss alerts (triggered by loss of ≥7 lbs in 1 week or ≥12 lbs in 1 month) occurred. No evidence of unhealthy weight loss behaviors was found; reasons for the triggers included scale calibration error (25% of the 185 weight loss alerts), another family member using the scale (10%), and other reasons (e.g., resumption of diet or exercise, illness, or return from vacation). In regard to adverse events, 19 events were reported by participants over the course of the study; none were believed to be related to the study.

## DISCUSSION

In a uniquely designed trial that enrolled and interacted with participants via the internet and provided monetary incentives for weight maintenance or additional weight loss, successful weight loss maintenance occurred across both incentive arms and the control arm. These incentive strategies had not been tested previously for weight loss maintenance. Both direct and lottery-based incentives led to additional weight loss over the 6 months these incentives were available, but not to a degree that was significantly different than text message feedback based on daily weighing. That all arms maintained weight loss likely reflects a beneficial impact of the daily weighing with feedback, accountability from the in-person weight measurements every 3 months, ongoing participation in the WW program, and the fact that the sample was comprised of a group of volunteer participants who had achieved significant initial weight loss and likely were highly motivated^18^. Other clinical trials have shown weight regain among participants after significant weight loss so it seems unlikely that the observed success of all three groups is due to selection alone^7^.

Whereas our study focused on the impact of financial incentives on weight loss maintenance, several previous studies have shown the benefit of financial incentives on initial weight loss^19^. One study found that participants offered US$14 per percentage point of weight loss lost more weight over 3 months than participants offered US$7 per percentage point and control participants^20^. Previous studies confirmed that weight loss could be enhanced by using deposit contracts, whereby participants in a weight loss program made up-front payments and received a percentage up to the full amount back depending on the amount of weight loss^21,22^. In a 24-week intervention, participants who received incentives as a group (US$500 per month divided among five participants) for meeting weight loss goals lost more weight (4.8 kg) than participants who received individual (US$100 per month) incentives (1.7 kg, *p* = 0.008) or control participants (0.5 kg, *p* < 0.001);^23^ at 36 weeks, group incentive participants maintained greater weight loss than control but not individual incentive participants. Another 3-arm study found that participants receiving deposit contract or lottery-based incentives over 16 weeks lost more weight (6.4 and 6.0 kg, respectively) than control participants (1.8 kg), but after cessation of incentives, differences were no longer present at 7 months^8^.

The financial incentives we tested were based on several strategies from behavioral economics. First, research has shown that even small rewards or punishments have strong incentive value if they occur immediately^24,25^, so qualifying participants received immediate feedback about their earnings. We provided the payouts only every 3 months in order to avoid the “peanuts effect” from small payments^26^ and so that accumulating balances would create an endowment effect whereby participants would not want to lose their accumulated winnings and loss aversion might provide further motivation to maintain or keep losing weight until the next in-person weigh-in^27^. The immediacy of the rewards, however, may have been tempered by the requirement of in-person weight measurement every 3 months to receive payouts. Second, avoidance of regret is a powerful influence in decision making under risk^28^, which is the reasoning behind giving feedback about what would have been won to incentive participants who did not reach goals. Third, data support that people are motivated by remembering past rewards and contemplating future rewards^29^ and are particularly attracted to small probabilities of large rewards;^30^ therefore, the lottery was designed to offer frequent small payoffs (roughly a 1 in 5 chance at a US$10 reward) and infrequent large payoffs (a 1 in 100 chance at a US$100 reward). Fourth, lotteries also provide variable reinforcement, which has been demonstrated as more effective in reinforcing behavior than consistent reinforcement^9^.

The use of technology in this trial was both a strength and limitation. The use of a wireless scale and text messaging feedback is highly scalable, convenient for participants, and efficient for staff monitoring participant progress. The reliance on technology, however, may be a barrier for participants who do not own or are less savvy with the needed technology; for example, 17 participants required a replacement scale and 24 participants received a scale, but did not activate it in time to be enrolled in the study. Because of concerns regarding feasibility and cost for a national sample, a questionnaire was used to assess physical activity rather than accelerometer, but this may have overestimated more vigorous activity and underestimated sedentary activity^31^. Partnering with a national weight loss program facilitated enrollment and enhanced geographic generalizability, but also may have limited generalizability because participants were predominantly white females. The platform for delivering the intervention, however, can be easily connected with any weight loss program by simply providing an electronic link. The frequent feedback was designed to increase motivation in participants who meet their goals but might frustrate participants who are not meeting goals. Further, the daily text messaging platform allowed immediate feedback to enhance adherence, but the actual payouts were every 3 months and dependent on maintaining weight in the interim.

In a pragmatic study that enrolled participants using a passive system for electronic monitoring of weights, we were able to briskly enroll and follow participants in a behavioral program that resulted in successful weight loss maintenance. However, adding direct or lottery-based incentives to the control condition of daily weighing with text messaging feedback and in-person weigh-in accountability every 3 months plus the foundation of the WW program did not provide clear additional benefit despite its additional cost. The electronic platform for intervention delivery is a viable option for large-scale weight loss programs that might, for example, be deployed in work place settings that desire efficient strategies to enhance maintenance of weight loss. Future research might investigate incentives that become more potent over time such as increasing dollar amounts or might pair financial incentives with competitions or other social platforms that could enhance motivation synergistically.

## Electronic supplementary material

Text messaging wording

eFigure 4. Mean Weight Change in kg at 6 Months in All Arms Combined by At-home Self-Weighing
