# Supplementary material for: Financial incentive strategies for maintenance of weight loss: results from an internet-based randomized controlled trial
Source: Nutr Diabetes. 2018 May 25;8:33. doi: 10.1038/s41387-018-0036-y (PMC5968035; doi:10.1038/s41387-018-0036-y)
Supplement: Supplementary file 1 — Text messaging wording [file 41387_2018_36_MOESM1_ESM.docx]

Supplement 1. Text messaging wording

Control:

If met goal: Congratulations - you met your weight loss goal yesterday! Continue to weigh in daily in order to track your progress.

If didn’t meet goal: Unfortunately, you either did not weigh in or you did not meet your weight loss goal yesterday. Try not to get discouraged and please weigh in again today.

Direct:

If met goal: Congratulations - you met your weight loss goal yesterday! For this achievement, $2.80 will be added to your winnings balance today [date]! Continue to weigh in daily for the chance to earn more money.

If didn’t meet goal: Unfortunately, you either did not weigh in or you did not meet your weight loss goal yesterday. Try not to get discouraged and please weigh in again today.

Lottery:

If met goal & won lottery: Congratulations - you met your weight loss goal yesterday and won the lottery on [date]! The number drawn was [winning number] and your number is [participant’s lottery number]. You won [$amount]. Continue to weigh in daily for the chance to win more money.

If met goal but didn’t win lottery: Congratulations - you met your weight loss goal yesterday but none of your lucky numbers were drawn on [date]. Continue to weigh in each day and you will have the chance to win the daily lottery.

If didn’t meet goal & would have won lottery: Unfortunately, you either did not weigh in or did not meet your weight loss goal yesterday. The number drawn was [winning number] and your number is [participant’s lottery number]. Had you met your goal, you would have won [$amount] on [date]. Try not to get discouraged and please weigh in again today.

If didn’t meet goal & would not have won lottery: When the lottery was run on [date], you either did not weigh-in or you didn't meet your goal the day before. Had you weighed in or met your goal, you would have been eligible for the lottery on [date]. Please try not to get discouraged and weigh in again today.
