# Supplementary material for: Financial incentive strategies for maintenance of weight loss: results from an internet-based randomized controlled trial
Source: Nutr Diabetes. 2018 May 25;8:33. doi: 10.1038/s41387-018-0036-y (PMC5968035; doi:10.1038/s41387-018-0036-y)
Supplement: Supplementary file 2 — eFigure 4. Mean Weight Change in kg at 6 Months in All Arms Combined by At-home Self-Weighing [file 41387_2018_36_MOESM2_ESM.docx]

**eFigure 4. Mean Weight Change in kg at 6 Months in All Arms Combined by At-home Self-Weighing Frequency**
